# Gauging Quince Phytonutrients and Its 4% Emulgel Effect on Amplifying Facial Skin Moisturizing Potential

**DOI:** 10.3390/gels9120934

**Published:** 2023-11-28

**Authors:** Tanzila Khiljee, Naveed Akhtar, Sonia Khiljee, Bushra Khiljee, Hafiz Majid Rasheed, Siddique Akber Ansari, Hamad M. Alkahtani, Irfan Aamer Ansari

**Affiliations:** 1 Faculty of Pharmacy and Alternative Medicines, The Islamia University of Bahawalpur, Punjab 63100, Pakistan; tk_pharmacist786@yahoo.com (T.K.); nakhtar567@hotmail.com (N.A.); 2 Shahida Islam College of Pharmacy, Shahida Islam Medical Complex, Lodhran 59320, Pakistan; bushra_khiljee@yahoo.com; 3 Faculty of Pharmacy, The University of Lahore, Lahore 54590, Pakistan; drmajid.rph@gmail.com; 4 Department of Pharmaceutical Chemistry, College of Pharmacy, King Saud University, Riyadh 11451, Saudi Arabia; sansari@ksu.edu.sa (S.A.A.); ahamad@ksu.edu.sa (H.M.A.); 5 Department of Drug Science and Technology, University of Turin, 10124 Turin, Italy; irfanaamer.ansari@unito.it

**Keywords:** quince, in vivo assessment, moisturizer, chlorogenic acid, facial skin resilience

## Abstract

Background: The aim of this study was to evaluate the moisturizing efficacy of quince fruit, used in folk medicine. For this purpose, the phytoconstituents of *Cydonia oblonga* fruit extract, like phenolics and flavonoids, were determined. A stable cosmetic emulgel containing 4% *Cydonia oblonga* fresh fruit extract was formulated and subjected to in vivo evaluation compared with a control. Materials and Methods: *Cydonia oblonga* fresh fruit extract was evaluated for tyrosinase activity and phenolic and flavonoid content. A stable emulgel containing 4% *Cydonia oblonga* fresh fruit extract was formulated and tested in a skin irritation test. After this, in vivo tests of erythema, moisture, sebum, and skin elasticity were conducted. The in vivo evaluation was a randomized and single-blind study. Thirteen healthy female volunteers were selected for a three-month study period. Results: *Cydonia oblonga* fruit extract showed good phenolic and flavonoid content, which was associated with its good antioxidant and tyrosinase-inhibiting activity. *Cydonia oblonga* containing the emulgel showed a reduction in sebum and erythema, while the elasticity and moisture content showed increments in their levels after the three-month application of the formulation. The fruit contains chlorogenic acid and many sugars, which might account for its anti-inflammatory and sebum reduction effects; it is also capable of enhancing the skin’s hydration level and decreasing skin sagging by enhancing its elasticity. Conclusion: The emulgel loaded with *Cydonia oblonga* fresh fruit extract is verified regarding its folklore status as a moisturizing agent that enhances the facial skin cells’ resilience potential.

## 1. Introduction

Moisturizers work by reducing trans-epidermal water loss (TEWL) by forming a layer around the stratum corneum. As a result, the stratum corneum becomes hydrated and soft. Many factors affect the skin’s hydration level, like environmental factors, diet and aging, etc. As the age increases, the skin loses the capacity to maintain its hydration level. Therefore, moisturizers from outside sources are required to increase skin health [1,2,3]. Asian women utilize many moisturizing agents found in the household, like honey, aloe vera, coconut oil, olive oil, different fruit pastes and fruit extracts. Many household moisturizers are available naturally, such as rose water, which helps to maintain skin tone and has been used traditionally for centuries. Among natural moisturizers, many botanical extracts have been widely investigated for their moisturizing effects, such as aloe vera, comfrey root and Avena sativa [4].

The Rosacea family is famous for its nutritional and delicious fruits. The Rosacea family genus Cydonia has one fruit named quince, which is commonly used in the kitchen as the main ingredient in different desserts. Furthermore, it also has many medicinal uses, such as in asthma and irritable bowel disease, as well as anticancer, antimicrobial, antifungal, anti-inflammatory, anti-erythematic, antiallergic, anti-ulcerative and wound-healing effects. *Cydonia oblonga* mucilage is used for wounds and burns. *Cydonia oblonga* is a native fruit of Iran, but it is also now cultivated around the world. It is reported in Iranian traditional medicine for a variety of diseases. Its seeds are soaked in water, which causes them to swell, and then a mucilaginous mass is obtained, which is effective for wounds. This mucilage has been supposed to increase the healing potential of skin in wounds. In previous studies, different creams have been compared with quince seed and it has shown better results than other creams. Moreover, mucilage used in cosmetics as a skin protectant. It is also commonly used as an antiaging agent as it tightens the skin and increases the skin’s resilience potential. Women use it to remove scars, improve skin sagging and reduce wrinkles, using a homemade herbal mixture. Traditionally, women used its gel as a facial moisturizer. It consists of mucilage, pectin, tannins, fatty oils and vitamins. The high mucilage content combines essential oils and fats with water. It is reported to be a good source of phenolic and flavonoid compounds and also exhibits good anti-tyrosinase activity [5,6,7,8].

Due to its rich phytonutrients, *Cydonia oblonga* extract has shown good moisturizing and soothing effects on the skin. The incorporation of herbal extracts in pharmaceutical dosage forms has been very useful in the cosmetic industry. Controlled clinical trials on these herbal cosmetics help to identify any possible adverse effects. 

The usage of botanical extracts in pharmaceutical topical dosage forms is not a new development. Many evidence-based plant extracts have been tested for their efficacy in laboratories and have been proven to be safe and effective [9]. Plant-based topical formulations have proven to be a promising source of therapeutic agents. Many topical formulations have been tested, but the emulgel formulation has proven to enable a high drug-loading capacity [10]. An emulgel is a formulation that contains both emulsion and gel characteristics. Due to its gel nature, the viscosity of the formulation is enhanced, causing greater adherence to the skin, which increases the staying potential of the topical formulation. Nowadays, a number of topical formulations are prepared in emulgel formulations. However, no research study has been reported yet in the context of a *Cydonia oblonga*-loaded topical emulgel formulation and its in vivo efficacy on human facial skin. Although oral use has proven health benefits, the current study was designed to validate the effectiveness of topical use as well. The cosmetic emulgel has proven to be a very successful dosage form for this fruit.

The aim of the current study was to determine the phytoconstituents of *Cydonia oblonga*, particularly flavonoid and phenolic content, as well as its anti-tyrosinase activity. Moreover, its in vivo efficacy on skin erythema, sebum, moisture and elasticity were determined on thirteen female volunteers along with controls on facial skin for a three-month study period in order to validate its use in folklore.

## 2. Result and Discussion

The *Cydonia oblonga* fruit is reported to be a rich source of polyphenolic constituents like chlorogenic acid, quercetin, caffeic acid and kaempferol [11,12,13]. In the current study, the total phenolic content of *Cydonia oblonga* (fresh peel and pulp) was 25.48 ± 1.52 (mg GAE/g of sample) and the total flavonoid content of *Cydonia oblonga* (fresh peel and pulp) was 14.76 ± 0.84. The current findings are in consistent with previous results in which it also showed a good amount of phenolic and flavonoid compounds [12,13,14]. Moreover, it was reported that the total phenolic content of the *Cydonia oblonga* fruit peel was higher whether it was in a lipophilic extract or hydrophilic extract, and the TPC of the *Cydonia oblonga* peel and pulp was greater than that in the seeds [15]. Furthermore, the hydro-alcoholic fruit extract of *Cydonia oblonga* showed good tyrosinase-inhibiting activity. Anti-tyrosinase activity is strongly related to the phenolic and flavonoid content of the extract [16,17].

In the current study, the emulgel loaded with the *Cydonia oblonga* fruit extract and the control emulgel subjected to the skin patch test did not cause any itching on the facial skin [18]. Thus, the tested emulgel is safe and shows no adverse effects. 

In human skin, melanocytes produce melanin, which is responsible for the skin color. The production of melanin is controlled by three enzymes, named tyrosinase, tyrosinase-related protein-1 and tyrosinase-related protein-2. Plant secondary metabolites like phenolics and flavonoids directly affect the melanin-producing enzymes [19]. As the *Cydonia oblonga* fruit extract showed high total phenolic and flavonoid content, more studies are needed to assess its effects on melanin in skin. 

*Cydonia oblonga* has been documented in Iranian traditional medicine as a remedy for wounds, burns, inflammation and skin allergies. In the present study, the topical emulgel containing *Cydonia oblonga* fruit extract also demonstrated its anti-erythematic potential, which was obvious from the results. The formulation showed a marked reduction in skin inflammation. This was due to the presence of chlorogenic acids, such as caffeoylquinic acid and dicaffeolyquinic acid, in the *Cydonia oblonga* fruit extract, which provide its strong antioxidant activity and reduce inflammation. Some previous studies also support this traditional use: in one study, its mucilage reduced allergic skin conditions, and in another research study, it was used as a healing agent for wounds and burns in mice [8,20]. Moreover, in a study, it was proven that caffeoylquinic acid, dicaffeoylquinic acid and vicenin play a role in prostaglandin inhibition, causing a reduction in inflammation [21]. The *Cydonia oblonga* fruit contains caffeoylquinic acid, dicaffeoylquinic acid and vicenin, which contribute to its anti-inflammatory potential against sun-induced inflammation.

Aging causes skin sagging and wrinkles as the skin loses its elasticity. Similarly, as the skin becomes dehydrated, it develops wrinkles. Thus, external moisturizers are necessary to keep the skin hydrated and increase its elasticity. Externally, *Cydonia oblonga* mucilage has been used by many people as a moisturizer. In the present study, the *Cydonia oblonga*-loaded emulgel increased skin elasticity and moisture content. The *Cydonia oblonga* fruit contains caffeoylquinic acid, dicaffeoylquinic acid, vitamins and sugars (rhamnose, mannose, D-glucose, L-arabinose and galactose mono-saccharides), which help in collagen fiber regeneration, thus contributing to skin elasticity and hydration [22]. 

The current study also showed a reduction in the sebum level of the skin with the application of the *Cydonia oblonga*-loaded emulgel compared to the control. A homemade topical mixture of *Cydonia oblonga* is commonly used by women as an acne remedy. The *Cydonia oblonga* fruit contains many acids (chlorogenic acid, ascorbic acid, citric acid, malic acid, shikimic acid and fumaric acid), which might be helpful in treating acne by reducing the pH of the skin [22].

### 2.1. Tyrosinase Inhibitory Activity (TIA)

Results showed good activity of tyrosinase inhibition at a concentration of 1 mg/mL, with 90% mushroom tyrosinase inhibition, in contrast with kojic acid (as control), which showed 99% activity. These results showed that the aqueous methanolic fruit extract displayed the highest tyrosinase inhibitory activity, and the reading was 33 ± 0.56 (Mean ± SEM, *n* = 3) % inhibition.

### 2.2. Total Phenolic Content (TPC)

The value of the total phenolic content in the *Cydonia oblonga* extract was 25.48 ± 1.52 (Mean ± SEM, *n* = 3) µg GAE/mg.

### 2.3. Total Flavonoid Content (TFC)

The value of the total flavonoid content was 14.76 ± 0.84 (Mean ± SEM, *n* = 3) µg QE/mg.

It is evident from the results of the phytochemical examination that the *Cydonia oblonga* fruit extract achieved the largest value of TFC and TPC, as well as tyrosinase-inhibiting activity.

### 2.4. Formulation Development

The formulations (active and control) were found to be stable after stability studies in a 12th week timeframe. The color of the active formulation was light brown, and the color of the base was white, which remained the same throughout the studied time period of 12 weeks.

All developed formulations, i.e., the active topical emulgel and control topical emulgel, were stable and completed the stability testing for a three-month time period under varying conditions of temperature and humidity.

### 2.5. In Vivo Examinations

#### 2.5.1. Skin Patch Test

**Figure 1:**
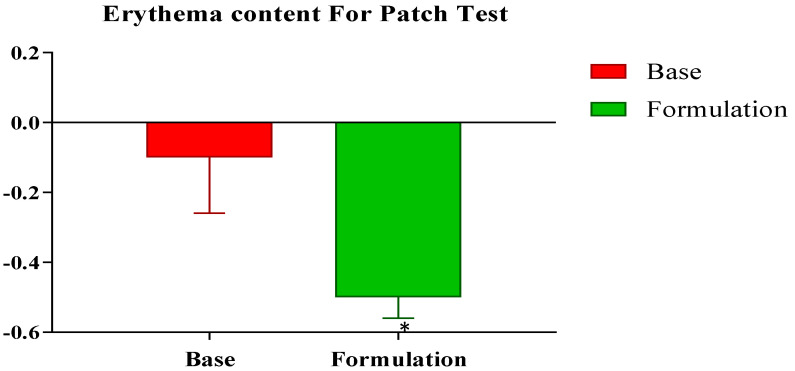
Percentage changes in skin erythema level for active formulation containing *Cydonia oblonga* extract against control after 48 h. * indicates statistically significant difference (*p* < 0.05).

The results of the skin patch test did not reveal any itching/inflammation in any participant in the three groups within 48 h of the utilization of the control and active emulgels (Figure 1). For both the control and active emulgels, the observations indicated that both could be applied safely to the participants for in vivo evaluations.

#### 2.5.2. Assessment of Skin Erythema

**Figure 2:**
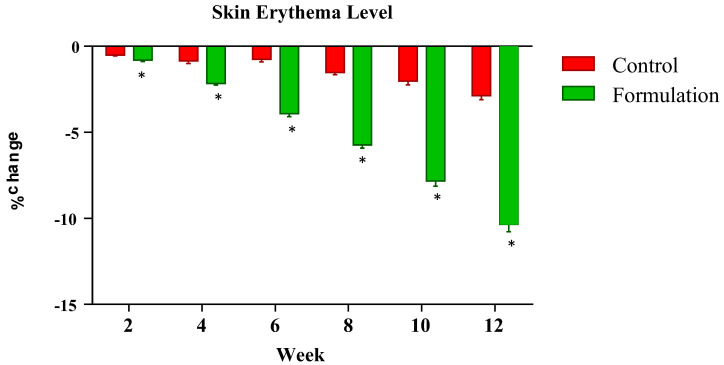
Percentage change in skin erythema level after the use of active formulation versus control. * indicates statistically significant difference (*p* < 0.05).

The erythema index was measured at systematic time intervals of zero time and the 2nd, 4th, 6th, 8th and 12th weeks of the study period. Changes in the erythema level of the tested formulation and control formulation were noted. Results showed that the active formulation caused a systematic decline in the erythema readings of participants during the study period. The mean percentage changes ranged from −0.81% in the 2nd week to −10.35% in the 12th week. Meanwhile, the control showed an abrupt fall in readings, being −0.52% in the 2nd week and −2.87% in the 12th week (Figure 2). Statistical analysis using ANOVA with a 5% level of significance showed significant decrease in erythema as compared to the control. When applying the LSD, the active formulation showed a significant effect during the study period as compared to the control. Furthermore, the PST showed that significant changes were observed between the active formulation and control with the passage of time.

#### 2.5.3. Assessment of Skin Moisture

**Figure 3:**
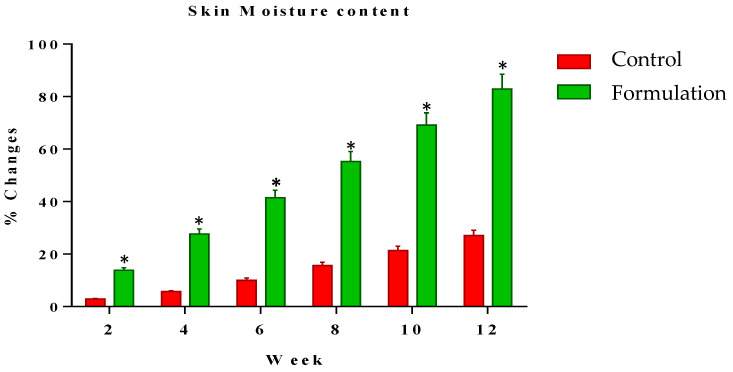
Percentage change in moisture level of skin after the use of active formulation versus control. * indicates statistically significant difference (*p* < 0.05).

Skin hydration was measured at systematic time intervals of zero time and the 2nd, 4th, 6th, 8th and 12th weeks of the study period. Changes in the skin’s hydration level with the tested formulation and control formulation were noted. The moisture level results were 5.13% in the 2nd week and 56.49% in the 12th week of study. Meanwhile, the control (base) values were 1.96% in the 2nd week to 10.76% in the 12th week of study (Figure 3). Statistical analysis using ANOVA with a 5% level of significance showed an increment in the skin’s hydration level that was significant as compared to the control. When applying the LSD, the active formulation showed a significant effect during the study period as compared to the control. Furthermore, the PST showed that significant changes were observed between the active formulation and control with the passage of time.

#### 2.5.4. Assessment of Skin Elasticity

**Figure 4:**
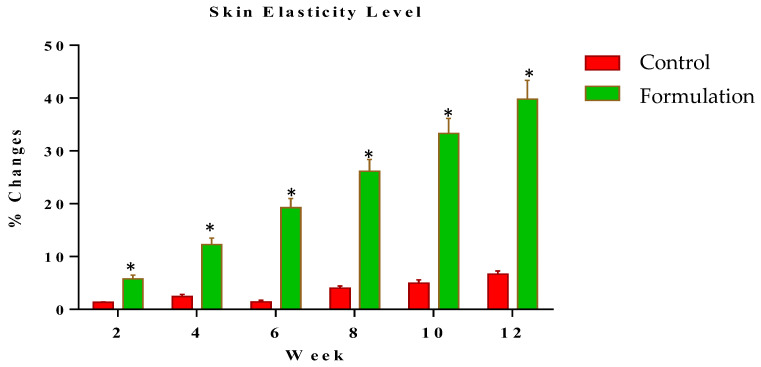
Percentage change in elasticity of skin after the use of active formulation versus control. * indicates statistically significant difference (*p* < 0.05).

The elasticity of skin was measured at systematic time intervals of zero time and the 2nd, 4th, 6th, 8th and 12th weeks of the study period. Changes in skin elasticity with the tested formulation and control formulation were noted. The result of skin elasticity at the 2nd week was 5.75% and further increased to 39.81% at the 12th week. Meanwhile, for the control, the result at the 2nd week was 1.36%, increasing to 6.67% in the 12th week of study (Figure 4). Statistical analysis using ANOVA with a 5% level of significance showed that skin elasticity showed significant results as compared to the control. When applying the LSD, the active formulation showed a significant effect during the study period as compared to the control. Furthermore, the PST showed that significant changes were observed between the active formulation and control with the passage of time.

#### 2.5.5. Assessment of Skin Sebum

**Figure 5:**
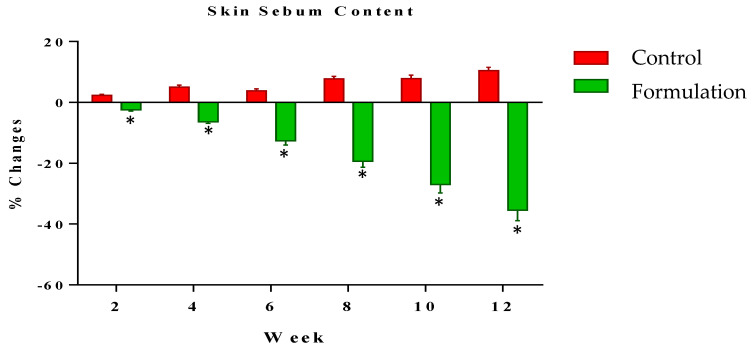
Percentage change in sebum level of skin after the use of active formulation versus control. * indicates statistically significant difference (*p* < 0.05).

Skin sebum levels were measured at systematic time intervals of zero time and the 2nd, 4th, 6th, 8th and 12th weeks of the study period. Sebum level results showed a gradual reduction in the readings for the whole study duration of three months. The reading was −2.45% in the 2nd week and further declined to −35.45% in the 12th week. On the other hand, the control emulgel showed an increment in the readings from the 2nd week (2.35%) to the 12th week (10.44%), as described in Figure 5. Statistical analysis using ANOVA with a 5% level of significance showed that the active formulation reduced the sebum level significantly as compared to the control. When applying the LSD, the active formulation showed a significant effect during the study period as compared to the control. Furthermore, the PST showed that significant changes were observed between the active formulation and control with the passage of time.

## 3. Conclusions

The present research work validates the folkloric use of *Cydonia oblonga*. It has been proven that the fresh fruit extract of *Cydonia oblonga* is an excellent source of polyphenols and flavonoids, as evidenced by its anti-tyrosinase activity. A stable 4% emulgel containing *Cydonia oblonga* fruit extract was formulated. The in vivo evaluation of the tested formulation showed significant results compared to the control. The emulgel containing *Cydonia oblonga* showed a reduction in erythema and sebum levels, making it suitable as an anti-inflammatory agent. It also showed a good moisturizing effect as it controlled moisture loss from the facial skin, allowing a marked increment in skin moisture and elasticity. The formulation could be promising for cosmetics designed to enhance the facial skin’s resilience due to its good moisturizing potential. Furthermore, it is free from any side effects. The current study validates the traditional topical use of *Cydonia oblonga* fruit for its anti-aging potential. Moreover, there is a need for further work on the anti-melanin activity of the formulation, as well as formulating this emulgel on an industrial level.

## 4. Material and Methods

### 4.1. Chemicals and Instruments

Liquid paraffin, propylene glycol and triethanolamine (Merck KGaA, Darmstadt, Germany). Methyl paraben (Acros Organics (Fisher Scientific, Waltham, MA, USA). Span 20, Carbopol 940 and Tween 20 (Sigma, St. Louis, MO, USA). All substances used were of analytical grade. Rotary evaporator (Heidolph, Co., Ltd., Kyoto, Japan), refrigerator (Dawlance, Lahore, Pakistan), hot incubator (Sanyo MIR-162, Osaka, Japan), mexameter, corneometer, sebumeter and elastometer (Courage + Khazaka Electronic GmbH, Köln, Germany).

### 4.2. Botanical Material

*Cydonia oblonga* fruit was obtained from Islamabad, Pakistan. The fresh fruit was identified and evaluated at the Department of Plant Sciences, Herbarium of Quaid-I-Azam University Islamabad, Pakistan, and the sample was assigned a voucher number (V# 1125). 

### 4.3. Preparation of Fresh Fruit Extract

The fresh fruit of *Cydonia oblonga* (one kg) was ground with distilled water in an electrical fruit juicer and analytical-grade methanol was added to the mixture to make the final volume up to one liter (*v*/*v* 70% hydro-methanolic extract). The mixture was macerated for three days with shaking after every 24 h. Then, the mixture was filtered with a muslin cloth and again filtered with filter paper (Whatman no. 20). This filtration process with filter paper was repeated three times. The obtained macerate was evaporated though a rotary evaporator to make the final condensed volume one fourth of the total volume. The extract was kept in an airtight closed glass flask and cooled at 4–8 °C before use [9].

### 4.4. Determination of Tyrosinase Inhibition Activity

Tyrosinase inhibitory analysis was performed according to Kim, with small alterations. Sixty units of enzyme, ten milliliters of the test compound and one hundred and fifty µL of buffer (50 mM of pH 6.8) were placed in every well and kept warm (in an incubator) for fifteen minutes at the temperature of 30 °C. Afterward, an incubation pre-reading at 480 nm was taken and 1 mM of substrate per well was further added. The mixtue was again incubated at the same conditions for thirty minutes and another reading was taken at 480 nm. Kojic acid was taken as a standard control. The result was observed by applying a formula. The IC50 was calculated by creating a stepwise dilution of the original concentration. Evaluations were carried out in triplicate. The following formula was used [23].

$$\mathrm{Inhibition}\;(\%)\;=100-\left(\frac{\mathrm{Absorbance}\;\mathrm{of}\;\mathrm{text}\;\mathrm{compound}}{\mathrm{Absorbance}\;\mathrm{of}\;\mathrm{control}}\right)\times 100$$

In the above equation, text compound denotes the tested extract sample and control denotes the standard.

### 4.5. Determination of Total Phenolic Content

The total phenolic content (TPC) of the sample extract was calculated by using the colorimetric FCR technique as defined by Wolfe et al., with some alterations. Ten µL of 10% diluted Folin–Ciocalteu reagent (FCR) was combined with one hundred µL of trial solution and kept warm (incubated) for ten minutes, followed by the addition of ninety µL of 15% (*w*/*v*) Na_2_CO_3_ aqueous solution. This concoction was incubated for an additional ninety minutes at 37 °C. Absorbance was calculated at 750 nm. Both the positive (gallic acid) and negative controls were involved. The TPC was calculated by constructing a calibration curve in the range of zero–one hundred µg, and results were expressed as mg gallic acid equivalent per gram of dry extract (mg of GAE/g of DE). Evaluations were carried out in triplicate [24].

### 4.6. Determination of Total Flavonoid Content

The total flavonoid content (TFC) was calculated by the improved colorimetric process. The calibration curve was obtained using quercetin as a reference standard, with one mg/mL of methanol, ranging from zero to one hundred µg. All solutions were prepared in methanol. One hundred µL of sample solution was combined with twenty-five µL of 1% sodium nitrite solution and left to stand for five minutes, monitored by adding ten µL of 10% AlCl_3_ solution and left again to adjust for five minutes. As a final point, thirty-five µL of 4% solution of NaOH was further added and the mixture was diluted with thirty µL of methanol. Absorbance was calculated at 510 nm. Total flavonoid content was measured by using the equation of the calibration curve and indicated as mg quercetin equivalent per gram of dry extract (mg of QE/g of DE). Evaluations were carried out in triplicate [24].

### 4.7. Preparation of 4% Cydonia oblonga Cosmetic Emulgel

Emulgel was formulated by the addition of the fresh fruit methanolic extract, denoted as the active emulgel (loaded with fruit extract) and control emulgel (without fruit extract). The aqueous phase (Tween 20, propylene glycol, methyl paraben and distilled water) of the emulsion was formed by heating to 75 ± 1 °C. The *Cydonia oblonga* fresh fruit extract was added to the aqueous phase. The oily phase (paraffin oil and Span 20) was heated to the same temperature as the aqueous phase. Then, the aqueous phase was combined with the oily phase with slow continuous shaking to produce an oil-in-water (*o*/*w*) emulsion. Then, 1.5 g of Carbopol 940 was added in distilled water to increase the final volume to 100 mL. A dispersion was obtained in which a solution of triethanolamine was added dropwise until it reached a pH of 5.5–6.5. At the end, the *o*/*w* emulsion was added to the gel under constant shaking for 15–20 min at the speed of 2000 rpm using a digital homogenizer; then, the speed of the digital homogenizer was reduced to 1000 rpm for five minutes and was again decreased to 500 rpm for five minutes to achieve the uniform mixing of the emulsion. It was then cooled at 25 °C. Throughout the mixing process, several drops of fruit oil were added in order to provide a pleasant odor to appeal to the female subjects. All developed formulations, i.e., the active topical emulgel and control topical emulgel, were subjected to stability studies for a three-month time period under varying conditions of temperature and humidity [25].

### 4.8. Non-Invasive In Vivo Analysis

#### 4.8.1. Study Protocol and Ethical Approval

The present study was a single-blind study designed for the comparison of the two developed emulgels, i.e., the active formulation with the *Cydonia oblonga* extract and other as the control (base without fruit extract). Thirteen female subjects were recruited. The ages of the subjects were within the range of 20–32 years. Both developed formulations, active and control, were utilized in a patch test. After completing the patch test, each subject was provided with the two formulations (active formulation and control); the subjects were instructed regarding the application of the formulation on the face two times a day. Every subject was given a schedule of 2, 4, 8, 10 and 12 weeks. The present study was approved by the Pharmacy Research Ethics Committee (PREC) for in vivo studies (Ref. No. 46/S-2018-/PREC), the Islamia University of Bahawalpur, Pakistan, and it was conducted according to the international guiding principles of the Helsinki Declaration [25].

#### 4.8.2. Skin Patch Test

To determine the allergic potential of the developed formulations, i.e., the active and control emulgels, a skin patch test was performed. For this purpose, a patch on the forearm was marked with a size of 20 cm. The control emulgel was applied on the right arm and the active emulgel was applied on the left forearm. After application, the erythema level of the skin was measured with a mexameter after 48 h.

#### 4.8.3. Skin Erythema Level

The skin erythema level was determined by a mexameter. This instrument works on the principle of light reflection and absorption on the body. For erythema measurements, it operates at 568 nm and 660 nm for absorbed and reflected wavelengths, respectively [26].

#### 4.8.4. Skin Moisture Level

A corneometer was used for skin moisture level determination. When the probe was in contact with the skin, the moisture level was quantified by the capacity difference in the epidermis. All readings were performed in triplicate and their average value was taken [27].

#### 4.8.5. Elasticity of Skin

An elastometer was used to calculate the elasticity of the skin. The skin elasticity potential was measured with the help of a probe. Its main principle is to determine the flexibility of the stratum corneum under disturbances such as suction and stretching. All readings were performed in triplicate and their average value was taken.

#### 4.8.6. Sebum Content of Skin

A sebumeter was used to determine the skin sebum content. This device determines the oils present on the skin, including sebum. It has a plastic strip that changes upon contact with the skin; with sebum, it becomes translucent. The translucency is provided by a photocell and indicates the sebum content. All readings were performed in triplicate and the average value was taken.

### 4.9. Statistical Data Analysis

The percentage (%) variations in the individual readings of various parameters of the skin were calculated by using the following formula:

$$\mathrm{Percentage}\;\mathrm{change}\;(\%)=\left[\frac{\left(A-B\right)}{B}\right]\times 100$$

where

A = individual value of any parameter at 2nd, 4th, 6th, 8th, 10th and 12th week;

B = zero-hour value of this parameter.

The readings acquired for various skin parameters (erythema, elasticity, skin moisture and skin sebum) were described statistically via the SPSS, V 21.0 software. Analysis of variance (ANOVA) was used to evaluate the eventual alterations between different time intervals, and a paired sample t-test (PSTT) was used to describe any variance between the two developed emulgels (active and control). A post-hoc analysis was also performed through the Least Significant Difference (LSD) method, which computes “pairwise comparisons”, i.e., the smallest significant difference between two means. A difference was determined to be significant at a *p*-value less than 5% (*p* < 0.05).

## Data Availability

The data presented in this study are available in the article.
